# Cognitive empathy across the lifespan

**DOI:** 10.1111/dmcn.15263

**Published:** 2022-05-20

**Authors:** Liam Dorris, David Young, Jill Barlow, Karl Byrne, Robin Hoyle

**Affiliations:** ^1^ Paediatric Neurosciences Research Group Royal Hospital for Children Glasgow; ^2^ Institute of Health & Wellbeing, Medical Veterinary & Life Sciences University of Glasgow Glasgow; ^3^ Department of Mathematics and Statistics University of Strathclyde Glasgow; ^4^ Glasgow Science Centre, Pacific Quay Glasgow UK

## Abstract

**Aim:**

To describe the development of cognitive empathy across the lifespan from a very large cohort using a standardized measure of cognitive empathy ability.

**Method:**

Participants (*n*=4545, age bands <5y to >75y, 60% female) were a convenience sample recruited voluntarily from visitors to the Glasgow Science Centre in the UK, who completed the Reading the Mind in the Eyes Test.

**Results:**

When compared to preceding age groups, we found significant developmental gains in empathy ability in children aged 6 to 7 years (*p*=0.048, d=0.45) and again at 10 to 12 years (*p*=0.042, d=0.23), followed by a slight reduction in ability during adolescence (*p*=0.087, d=–0.18), and functional maturity in those aged 19 to 25 years (*p*=0.001, d=0.76). Cognitive empathy abilities remained relatively stable across adulthood but gradually declined in people over 65 years, with notable decline in males over 75 years (*p*=0.001, d=–0.98). Females performed better than males at all ages.

**Interpretation:**

Understanding developmental issues in cognitive empathy could influence approaches to moral and social education for children, and health and social care support for older people. Standardized cognitive empathy tests could also provide novel approaches in the early detection of developmental vulnerabilities in a range of neurological conditions, and within neuropsychiatric and neurodegenerative disorders in which cognitive empathy is known to be impaired.

**What this paper adds:**

- Cognitive empathy is a late‐developing ability and changes across the lifespan.
- Cognitive empathy increases during childhood but with potentially altered abilities during adolescence.
- Cognitive empathy matures during early adulthood and gradually declines in older age.
- There is a female advantage in cognitive empathy abilities.

## What this paper adds

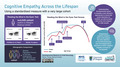

This original article is commented on by Baron‐Cohen et al. on pages 1440–1441 of this issue.

Video Podcast: https://youtu.be/QeTdLP75CKE

This article's abstract has been translated into Portuguese and Spanish.

Follow the links from the abstract to view the translations.

## Resumo

**Objetivo:**

Descrever o desenvolvimento da empatia cognitiva ao longo da vida de uma coorte muito grande usando uma medida padronizada da capacidade de empatia cognitiva.

**Método:**

Os participantes (n = 4545, faixas etárias de 75 anos, 60% do sexo feminino) formaram uma amostra de conveniência recrutada voluntariamente de visitantes do Glasgow Science Centre, no Reino Unido, que completaram o Reading the Mind in the Eyes Test.

**Resultados:**

Quando comparados às faixas etárias anteriores, encontramos ganhos significativos no desenvolvimento da capacidade de empatia em crianças de 6 a 7 anos (p = 0,048, d = 0,45) e novamente em 10 a 12 anos (p = 0,042, d = 0,23), seguido por uma ligeira redução na capacidade durante a adolescência (p = 0,087, d = –0,18) e maturidade funcional na faixa etária de 19 a 25 anos (p = 0,001, d = 0,76). As habilidades de empatia cognitiva permaneceram relativamente estáveis na idade adulta, mas diminuíram gradualmente em pessoas com mais de 65 anos, com declínio notável em homens com mais de 75 anos (p = 0,001, d = –0,98). As mulheres tiveram um desempenho melhor do que os homens em todas as idades.

**Interpretação:**

Compreender questões de desenvolvimento em empatia cognitiva pode influenciar abordagens de educação moral e social para crianças e apoio de saúde e assistência social para idosos. Testes padronizados de empatia cognitiva também podem fornecer novas abordagens na detecção precoce de vulnerabilidades de desenvolvimento em uma variedade de condições neurológicas e em distúrbios neuropsiquiátricos e neurodegenerativos nos quais a empatia cognitiva é sabidamente prejudicada.

## ResumenEmpatía cognitiva a lo largo de la vida

**Objetivo:**

Describir el desarrollo de la empatía cognitiva a lo largo de la vida de una cohorte muy grande utilizando una medida estandarizada de la capacidad de empatía cognitiva.

**Método:**

Los participantes (n = 4545, franjas de edad de 75 años, 60 % mujeres) fueron una muestra de conveniencia reclutada voluntariamente entre los visitantes del Centro de Ciencias de Glasgow en el Reino Unido, que completaron la Prueba de Lectura de la Mente en los Ojos.

**Resultados:**

En comparación con los grupos de edad anteriores, encontramos avances significativos en el desarrollo de la capacidad de empatía en niños de 6 a 7 años (p = 0,048, d = 0,45) y nuevamente a los 10 a 12 años (p = 0,042, d = 0,23), seguidos por una ligera reducción de la capacidad durante la adolescencia (p = 0,087, d = –0,18), y madurez funcional en los de 19 a 25 años (p = 0,001, d = 0,76). Las habilidades cognitivas de empatía se mantuvieron relativamente estables durante la edad adulta, pero disminuyeron gradualmente en personas mayores de 65 años, con una disminución notable en hombres mayores de 75 años (p = 0,001, d = ‐0,98). Las mujeres se desempeñaron mejor que los varones en todas las edades.

**Interpretación:**

Comprender los problemas de desarrollo en la empatía cognitiva podría influir en los enfoques de la educación moral y social para los niños y el apoyo de atención social y de salud para las personas mayores. Las pruebas estandarizadas de empatía cognitiva también podrían proporcionar enfoques novedosos en la detección temprana de vulnerabilidades del desarrollo en una variedad de afecciones neurológicas y dentro de los trastornos neuropsiquiátricos y neurodegenerativos en los que se sabe que la empatía cognitiva está alterada.

**Abbreviations**
GSC: Glasgow Science Centre
RMET: Reading the Mind in the Eyes Test
ToM: Theory of mind

Experimental psychologists, philosophers, and neuroscientists have long been interested in social cognitive abilities, that is how we solve the everyday challenge of understanding what another person may be thinking and/or feeling, and how we use those judgements to understand and predict their behaviour and navigate our way through the social environment. Despite the fundamental importance of these cognitive abilities for human survival and adaptation, remarkably little is known regarding their developmental trajectories across the lifespan.

There have been several terms used to describe the phenomena underpinning social cognition, with the aim of studying component processes involved in our understanding of empathy. In recent years the term ‘theory of mind’ (ToM) has become widely used in cognitive psychology and understood to describe the ability to infer and predict the intentions, thoughts, desires, intuitions, behavioural reactions, plans, and beliefs of other people,^1^ through an awareness that others have a mind with mental states, information, and motivations that may differ from one's own.^2^ For the purpose of the current study, we use the terms cognitive empathy and ToM as near synonyms, but acknowledge there is debate around how phenomenology involved in empathy research are defined.

There has also been increasing evidence for the distinction clinically and neurally between ‘cognitive’ and ‘affective’ empathy.^3^ ‘Cognitive empathy’ requires perceptual and cognitive neural circuits to identify and make inferences about the thoughts, intentions, and emotional states of others, whilst ‘affective empathy’ might be conceptualized as the ability to make inferences about what another individual is feeling and, crucially, to respond with the appropriate emotion. In this model it is plausible to correctly infer the intentions and beliefs of another but not to respond emotionally in a socially and morally normative manner. This dissociation in cognition and affect has been described in people with psychopathic traits, and in some individuals with autism spectrum disorders.^4^, ^5^

There is an increasing understanding of the neural basis of empathy; neuroimaging studies consistently implicate a network of brain regions including the medial prefrontal cortex, superior temporal sulcus, temporoparietal junction, and bilateral temporal poles.^6^, ^7^ There is also preliminary evidence suggesting cognitive and affective empathy are associated with specific neural architecture, with greater activation of the dorsolateral and ventromedial prefrontal cortex respectively.^8^, ^9^, ^10^

Much of the experimental consideration of ToM ability has been conducted with either very young children or with people with autism spectrum disorder, a condition where social cognition is severely affected. Widely used in autism spectrum disorder studies, the Reading the Mind in the Eyes Test (RMET)^11^ has also been used with typically developing controls, demonstrating that the test is sensitive in measuring social cognition across a wide ability range, and has been shown to discriminate between the first‐degree relatives of people with autism and typically developing controls.^12^ However, studies have typically been limited by small sample sizes often with around 25 participants in each experimental group.

In addition to autism spectrum disorder, ToM deficits are implicated in a range of other clinical disorders including schizophrenia^13^ and bipolar disorder,^14^ and in both children and adults with epilepsy.^15^ ToM deficits have also been found to mediate friendship quality and social integration after paediatric traumatic brain injury.^16^ Thus, ToM is a complex metacognitive process which is fundamental to social relationships and is impaired in a range of common neurological, neurodevelopmental, and psychiatric disorders.

The current study aimed to gather normative developmental data in a large, representative cohort of participants across the lifespan, using a well‐established measure of cognitive empathy ability. This information could be of value in understanding important cognitive developmental processes in humans, such as the most appropriate developmental age for children to be learning moral and social education in school, and informing our understanding of how empathy supports relationships and prosocial behaviour.

## METHOD

### Setting

Participants were visitors to the ‘Mind works’ exhibition at the Glasgow Science Centre (GSC; https://www.glasgowsciencecentre.org) over an 18‐month period. The GSC is a large facility providing science education and exhibitions with government subsidies to enable access, for example all schools in the Greater Glasgow urban area are able to send children for planned visits. Ethical approval was provided by the science committee of the GSC.

### Participants

The participants (*n*=4545) were prompted to provide their age band, sex, and postcode. Participants were then asked to select one of 12 age bands in years, before beginning the test (<6, 6–7, 8–9, 10–12, 13–18, 19–25, 26–35, 36–45, 46–55, 56–65, 66–75, >75). The largest age group by far was 36 to 45 years, accounting for almost a quarter of all participants (*n*=1094). Conversely, the over‐75s formed the smallest group, making up less than 1% of participants (*n*=43). Participant data was linked to routine demographic data collection for all visitors to the GSC.

The Scottish Index of Multiple Deprivation (https://simd.scot/#/simd2020/BTTTFTT/9/‐4.0000/55.9000/) uses postcodes to list every neighbourhood in Scotland in order of deprivation using a number of factors such as income rate and employment rate. The 6505 data zones are split into quintiles, five groups which are ordered from most deprived to least deprived (Q1–Q5).

**Figure 1:**
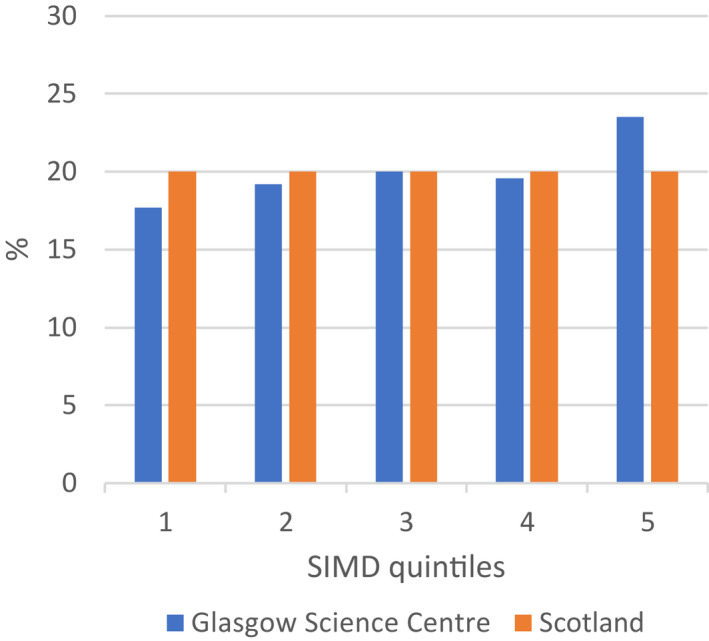
Deprivation (Scottish Index of Multiple Deprivation [SIMD]) quintiles for visitors to the Glasgow Science Centre compared to general population census data

Figure 1 shows the proportions of GSC visitors observed within each of the five Scottish Index of Multiple Deprivation quintiles. GSC visitors are slightly under‐representative of the most deprived Scottish Index of Multiple Deprivation data zones (17.7% vs 20% Q1) and slightly over‐represented by the least deprived data zones (23.5% vs 20% Q5) of Scottish Index of Multiple Deprivation areas.

**Figure 2:**
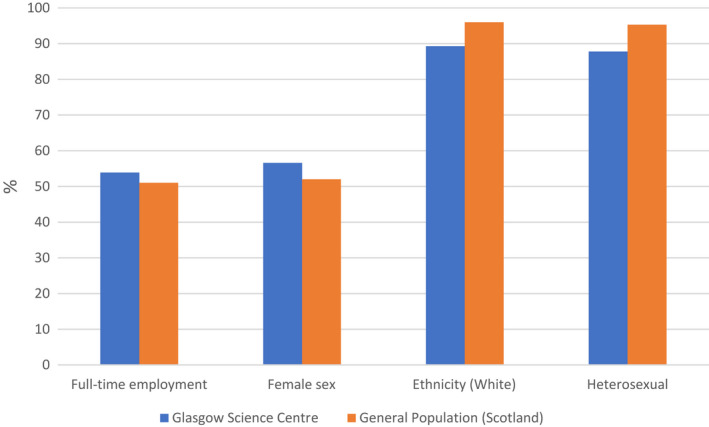
Demographic comparisons between Glasgow Science Centre visitors and general population census data for rates of full‐time employment, sex, ethnicity, and sexual orientation

We also compared GSC visitors on a range of demographic variables to the Scottish census data.^17^ There were more female visitors to the GSC than the general population ratio (61% vs 52%). GSC visitors were also slightly more diverse in terms of ethnicity (90% vs 95% White) and sexual identity (89% vs 95% heterosexual), but had similar rates of full‐time employment (52% vs 51%), see Figure 2.

### Measures

**Figure 3:**
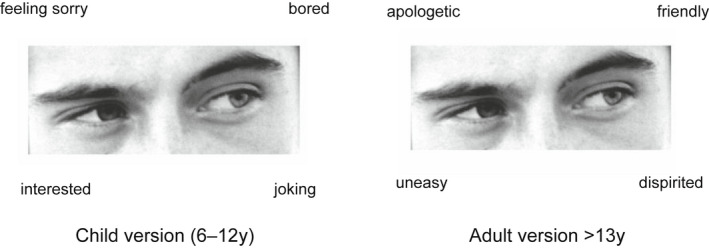
The Reading the Mind in the Eyes Test showing both child and adult versions

The RMET^11^ involves presentation of greyscale images of the eye region of the human face showing a range of emotions exhibited by actors (see Figure 3). The participants are then asked to choose one from four ‘mental state terms’ that ‘best describe what the person is thinking or feeling’. The mental state terms used in this test require linguistic and perceptual processing and are more complex tasks than identifying the six basic emotions as defined by Ekman et al.: happiness, sadness, fear, surprise, anger, and disgust.^18^ The RMET has been widely used in autism research and the original validation studies for the child and adult versions consisted of participants aged 6 to 13 years and 18 to 49 years respectively.^11^, ^19^ In order to reduce the time required to complete the test, participants were shown the first 21 out of 28 and 21 out of 36 of the original images from the child and adult versions of the test respectively. Children and adults completed versions with identical visual stimuli but the language used in the children's test was more straightforward. For example, ‘insisting’ in the adults' test was replaced by ‘making somebody do something’ in the children's test and similarly ‘decisive’ was substituted by ‘made up her mind’. Participants aged 12 years and under completed the child version of the test, while those aged 13 years and over completed the adult version. The RMET was digitized and presented on a 15‐inch computer screen built into a seated workstation located in the corner of a large exhibition space as part of a ‘Mindworks’ exhibition. The instructions were presented in a series of pages requiring completion in order to proceed to the test. Participants were instructed to choose which of four words that were presented in the corners of the visual stimuli best described what the person might be ‘thinking or feeling’. Younger children (<6y) were likely assisted by parent/carer in order to complete the RMET.

### Statistical analysis

The effects of age and sex on the RMET empathy score were investigated using multiple regression analysis.

To account for guessing in the multiple‐choice responses, scores were adjusted using the method proposed by Choppin:^20^ $\text{Adjusted percentage}=100\times \frac{p_{o}-p_{e}}{1-p_{e.}}$

where Po is the participant's proportion of correct answers and Pe is the proportion of correctly answered questions that would be expected by chance (i.e. 0.25). Therefore, anyone who correctly answered 100% of questions would remain with an adjusted score of 100%, whilst those who scored 25% in the test would have a corrected score of 0%. However, participants with a result less than 25% would have a negative score: this means they scored less than would be expected by chance. Adjusted scores were then analysed using a regression model which included age group, sex, and an age x sex interaction. All analyses were done using Minitab (version 18, Minitab LLC, State College, PA, USA) at a 5% significance level with the final model being chosen using stepwise selection. Where appropriate, a measure of effect size (Cohen's d) was reported to indicate the standardized difference between means alongside the significance level.

## RESULTS

There were 4545 participants (40% male) who completed the test included within the analyses. The final model found sex (*p*<0.001) and age (*p*<0.001) were highly significantly associated with cognitive empathy (model *R* ^2^=14.95%). There was no evidence of an interaction between age and sex (*p*=0.206). Those under 18 years and over 75 years had the poorest cognitive empathy skills (see Table 1).

**Table 1:** Summary data for each group by age and sex on the Reading the Mind in the Eyes Test

Age group, y	Sex	*n*	Mean (SD)	Median (range)
<6	Female Male	24 44	32 (24.44) 23 (22.96)	30.67 (−6.67, 74.67) 18.67 (−13.33, 81.33)
6–7	Female Male	49 46	40 (24.38) 34 (26.85)	37.33 (−6.67, 88) 37.33 (−13.33, 88)
8–9	Female Male	151 96	42 (26.25) 32 (21.57)	44.00 (−33.33, 88) 34.00 (−20, 74.67)
10–12	Female Male	311 232	44 (22.39) 43 (22.94)	44.00 (−20, 88) 44.00 (−13.33, 88)
13–18	Female Male	304 204	41 (22.95) 37 (23.01)	44.00 (−26.67, 94.67) 37.33 (−6.67, 88)
19–25	Female Male	318 143	57 (20.45) 55 (21.28)	59.33 (−6.67, 100) 56.00 (−6.67, 94.67)
26–35	Female Male	412 238	58 (19.75) 52 (24.10)	62.67 (−1.33, 94.67) 56.00 (−20, 100)
36–45	Female Male	666 428	60 (18.22) 58 (20.82)	62.67 (−1.33, 100) 62.67 (−6.67, 94.67)
46–55	Female Male	323 228	59 (18.83) 58 (20.33)	62.67 (−6.67, 100) 62.67 (−6.67, 94.67)
56–65	Female Male	113 75	56 (18.59) 52 (21.89)	56.00 (−1.33, 100) 56.00 (−6.67, 94.67)
66–75	Female Male	54 43	50 (21.57) 48 (21.70)	49.33 (−1.33, 88) 49.33 (−6.67, 81.33)
>75	Female Male	26 17	33 (22.71) 19 (22.73)	37.33 (−13.33, 69.33) 12.00 (−13.33, 62.67)

### Age effects

**Figure 4:**
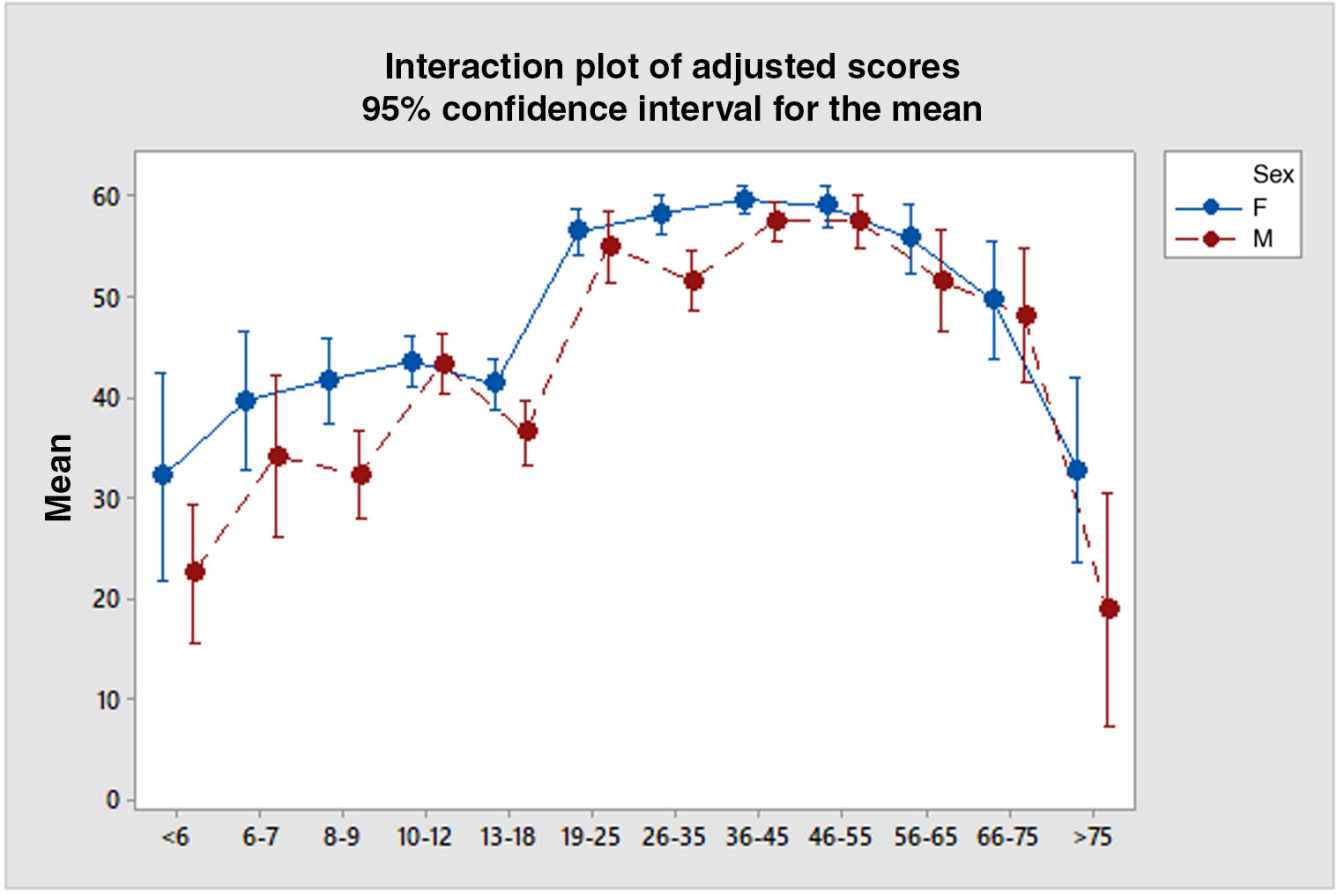
Adjusted mean scores by sex and age (y) on the Reading the Mind in the Eyes Test. [Correction added on 3 September 2022 after first online publication: In figure 4, the labelling of males/females has been amended to correct an error introduced by the production team]

Each of the 12 age bands were compared to the preceding age band to investigate the developmental periods during which cognitive empathy changes were most significant. There were three distinct periods of rapid development in childhood and early adulthood where performance was significantly greater than previous age bands: 6 to 7 years (*p*=0.048, d=0.45), 10 to 12 years (*p*=0.042, d=0.23), and 19 to 25 years (*p*=0.001, d=0.76). The most marked increase in cognitive empathy was between the 13‐ to 18‐years age group and the 19‐ to 25‐years age group, suggesting that late adolescence/early adulthood is a critical point at which cognitive empathy skills reach maturity (see Table 2). Performance remained stable across adulthood before declining in the seventh decade of life and very significantly declining in people aged over 75 years (*p*=0.001, d=0.98), see Figure 4.

**Table 2:** Theory of mind abilities comparing age bands

Age group, y	Difference	95% confidence interval	*p* *	Effect size
<6–6–7	11.11	3.31, 18.91	0.048	0.45
6–7 – 8–9	0.98	−4.98, 6.95	1.000	0.04
8–9 – 10–12	5.44	1.92, 8.96	0.042	0.23
10–12 – 13–18	−4.05	−6.82, −1.29	0.087	−0.18
13–18 – 19–25	16.66	13.88, 19.43	<0.001	0.76
19–25 – 26–35	−0.21	−2.75, 2.33	1.000	−0.01
26–35 – 36–45	2.99	1.03, 4.95	0.167	0.15
36–45 – 46–55	−0.35	−2.34, 1.63	1.000	−0.02
46–55 – 56–65	−4.34	−7.59, −1.09	0.400	−0.22
56–65 – 66–75	−5.12	−10.17, −0.06	0.746	−0.25
66–75 – >75	−21.73	−29.75, −13.71	<0.001	−0.98

* Adjusted for multiple comparisons.

### Sex differences

Females performed better than males at all ages (*p*<0.001); however, these differences were more prominent at several points across the lifespan. The largest discrepancy was found in the over 75 years group, where the female mean score was 13.9% higher than the male. However, this cohort was far smaller (*n*=43) than for other age bands with a wider confidence interval and therefore limits the precision of the population estimate. In contrast, in the 10‐ to 12‐year age group, females only performed 0.22% better than males, suggesting that this is a point in development where there is little difference in empathy ability (see Figure 4).

## DISCUSSION

Cognitive empathy develops in a nonlinear way across childhood and adolescence before maturing in the third decade of life. We also found that cognitive empathy abilities peak between the ages of 35 to 55 years, and slowly decline in older adults, particularly in males aged over 75 years.

Female sex was associated with enhanced ToM abilities at all stages of the lifespan. There continues to be debate in relation to whether this female advantage is a relatively fixed and stable trait difference, for example reflecting different hormonal effects such as in utero exposure to sex hormones, or whether there are sociocultural influences that may also differentially shape these abilities.^21^ Interestingly, there is some evidence for the neuroprotective effects of female sex in individuals suspected to have increased genetic loading for social cognitive deficits, for example female siblings of children with autism tend to score higher on empathy tests than typically developing IQ‐matched male controls, whilst scoring below female‐matched controls.^12^ Preliminary functional neuroimaging findings also indicate differential activation patterns between males and females on ToM tasks.^22^ The current findings might be considered alongside those related to sex‐related differences in cognitive abilities more generally, for example meta‐analytic studies confirm a cross‐cultural advantage in reading and writing, language, and verbal memory for females, whilst males tend to be significantly over‐represented in studies of those with superior maths and spatial reasoning ability.^21^ The important thing would seem to concern how we can understand potential sex differences in empathy and use this understanding to maximize developmental potential based on intrinsic cognitive style. Importantly, studies reporting sex‐based differences in cognitive ability reflect group differences, and many individuals will not conform to these sex‐linked cognitive profiles.

Another issue concerns how to promote the development of empathy abilities in children. The available evidence suggests that children show earlier awareness of mental states if their mothers/main carers explicitly talk about intentions, thoughts, and emotions^23^ and provide reasons when correcting misbehaviour,^24^ and that empathy development is also influenced by participation in pretend play, experience of storybook reading, and of talking with others about past experiences.^25^ There is also some evidence that siblings can accelerate ToM development,^24^ and that warm, sensitive, and responsive parenting is important for facilitating ToM development in children.^26^ The critical aspect of these interactions would appear to involve reflection and consideration of perspective and intention with a consistent caregiver. Understanding that prosocial behaviour has cognitive foundations might also actively influence early education approaches.

Interestingly, we found that late childhood (ages 10–12y) is a developmental period where children show greater empathy ability than adolescents. Therefore, late childhood is likely to be an ideal developmental period where moral, social, and relationship education could be particularly effective. Indeed, Weimer et al. found that adolescents who had more advanced ToM also showed more prosocial reasoning about conflict, which in turn mediated the relation with fewer serious behaviour problems in high school, after controlling for academic performance and sex.^27^

One of the more interesting findings in the current study concerns the moderate decline in ToM/empathy abilities during adolescence, where the 508 adolescent participants scored on average 10% lower than the 543 children aged 10 to 12 years. It is difficult to know why this may be, although there is some limited evidence for specific abilities such as facial recognition ability going ‘offline’ during periods of rapid development and rewiring within the prefrontal cortex.^28^ There is clearly a need to better understand the social cognitive neuroscience of adolescence in order to increase the engagement of young people, particularly those who may be at greater risk of social and educational exclusion or marginalization. Given the extent of neurological maturation and a corresponding increase in most intellectual abilities during adolescence, the current data on empathy are interesting. There is a need for more detailed studies of how cognition and learning are influenced during this important period of maturation by social and emotional stimuli.

Understanding the developmental trajectory of social cognition in typically developing children might also allow us to consider the development of children with known neurological insults or neurodevelopmental conditions. For example, recent studies suggest that autism may be significantly more common in people with cerebral palsy than in the general population, with reported incidences ranging from 2% to 30%, but that standard diagnostic methods are less sensitive for children with motor disability, particularly when this affects speech production.^29^, ^30^ One population‐based neuroimaging study reported autism in 30% of children with cerebral palsy, found across all imaging patterns but with increased incidence in those with white matter injury.^31^ There is also a growing number of studies examining the links between basal ganglia injuries and both cognitive dysfunction and neurodevelopmental disorders such as autism, posited to reflect disruptions in reciprocal neural networks between the basal ganglia and frontal cortex.^32^ There are likely to be a much larger number of children with cerebral palsy who do not have autism, but who may have vulnerability in social cognition and who may benefit from greater awareness and support for these difficulties in developing social relationships.

Another important finding concerns the decline in cognitive empathy shown in older age, particularly by older males in whom functioning by age 75 years was lower than the level attained by children less than 6 years of age. The relatively small sample size limits what we can conclude from the present findings, although age‐related decline has been previously reported.^33^

The implication of this data may be that many males may have difficulty in solving the complex task of recognizing other people's emotion and intention in later life. This could, under situations of high cognitive demand, lead to feelings of confusion or irritability. Whilst there is undoubtedly something important in the ‘wisdom of age’ hypothesis, and also in acknowledging that there will likely be significant individual variation in cognitive empathy ability, the potential implications of these data for older males seems important. It is also worth noting that ‘healthy’ ageing has been associated with reliable improvement in emotional well‐being and social functioning.^34^

Whilst the present study indicates that cognitive empathy ability may slowly reduce as a feature of typical ageing, there is also an increasing evidence bace establishing that empathy deficits are evident across neurodegenerative disorders.^35^ There is clearly potential utility in exploring whether standardized empathy tests could be used in detecting early cognitive changes in those with suspected neurodegenerative disorders, and which could also inform care planning approaches.

### Strengths and limitations

The main strengths of this study include the very large sample size and wide age range. Limitations included the use of a single short‐form measure of cognitive empathy, and the use of a community sample with no exclusion criterion, but the simplicity of the study also facilitated the participation of a large sample. We were not able to gather data on a range of variables such as physical health, IQ, family factors, sociocultural differences across time, and environmental factors that would allow us to build a more instructive model of what factors more precisely predict better cognitive empathy.

### Conclusion

These findings demonstrate how cognitive empathy improves across childhood, declines slightly in adolescence, matures in early adulthood remaining stable until late middle age and then declines, with a particularly steep decline in males over the age of 75 years. We confirm previous findings relating to the relative advantage shown by females in cognitive empathy abilities across the lifespan. The implications of this work seem significant; greater understanding of how children and adolescents understand and relate to others could allow improvements in educational approaches to moral and social development. The use of validated cognitive empathy tests could also become useful in the early detection of neurological disorders later in life.

## Data Availability

The data that support the findings of this study are available from the corresponding author upon reasonable request.
